# Electrochemical sensing of vitamin B_6_ (pyridoxine) by adapted carbon paste electrode

**DOI:** 10.1038/s41598-024-71341-2

**Published:** 2024-09-20

**Authors:** Ayah moustafa, Soha A. Abdel-Gawad, M. Shehata, Renad S. El-Kamel, Amany M. Fekry

**Affiliations:** 1 Chemistry Department, Faculty of Science, Cairo University, Giza, 12613 Egypt; 2 Faculty of Postgraduate Studies for Nanotechnology, Cairo University, Giza, 12613 Egypt

**Keywords:** Copper nanoparticles (CuNs), Vitamin B_6_, Phosphate buffer, Cyclic voltammetry, Urine, Carbon paste electrode (CPE), Chemistry, Nanoscience and technology

## Abstract

The recent investigation targets to use adapted carbon paste (CP) with copper nanoparticles (CuNs) operating in a phosphate buffer (PBS) medium with a pH range of 5.0–8.0, to synthesize a novel, susceptible, and simple electrochemical sensor for the detection of one of the most important drugs, vitamin B_6_. Copper (Cu) is one of the most three common essential trace elements found in the bodies of both humans and animals, along with iron and zinc for all crucial physiological and biochemical functions. Its properties, which are assessed using a variety of methods including scanning electron microscopy (SEM), cyclic voltammetry (CV), differential pulse voltammetry (DPV), and electrochemical impedance spectroscopy (EIS), have also drawn a lot of attention recently. We considered the effects of pH, buffer, scan rate, interference, and calibration curve. The susceptible electrode's linear calibration curve encompassed concentration values between 8.88 and 1000.0 µM. The calculated limits of detection and quantification were 32.12 and 107.0 µM, respectively. Furthermore, this method was established in real human urine samples and drug validation which have been shown satisfactory results for vitamin B_6_ detection.

## Introduction

There are two primary categories of nutrients: macronutrients and micronutrients. The body needs high quantities of macronutrients like proteins, carbohydrates, and fats since they give the human body molecules for metabolic and structural processes. In contrast, the body requires trace levels of micronutrients, which include vitamins and minerals, for optimal operation. An individual's metabolic activities and life cycle determine the need for micronutrients. Although our body needs only a small amount of micronutrients (in mg or μg), they are considered as significant as macronutrients. They are necessary for the body to develop. Since our bodies are unable to synthesize micronutrients, they must be obtained at appropriate levels from the diet. If their supply is insufficient, this can lead to a host of diseases. Vitamins are essential for the body's normal functioning and development. Two main categories can be used to classify vitamins: fat-soluble and water-soluble. While vitamins A, D, E, and K are fat-soluble, vitamins B_1_, B_2_, B_3_ (niacin), B_5_ (pantothenic acid), B_6_ (pyridoxine), B_7_ (biotin), B_9_ (folate), B_12_ (cyanocobalamin), and C (ascorbic acid) are water-soluble. Our study will be concerned with vitamin B_6_ (Pyridoxine), which belongs to the B-complex group of vitamins; it is a crucial co-factor for various biochemical processes that control fundamental cellular metabolism^1^. Vitamin B_6_ is a necessary food component for a well-balanced diet, which is also relatively abundant in many foods^2^. It proceeds as a coenzyme in several metabolic transformations in biological systems, including amino acid metabolism, transculturation, and glycogen phosphorylation^3^. It acts as an antioxidant, red blood cell formation, and haemoglobin production^4^. It may help in treating PMS symptoms^5^ and nausea during pregnancy^6^, may boost mood by alleviating depression symptoms^7–10^, may be used to treat rheumatoid arthritis-related inflammation^11–14^, promote eye health for avoiding eye diseases^15–19^, It also lowers the risk of Alzheimer's^20–22^, heart disease^23–26^ and cancer risks^27,28^. From the up mentioned we found that Vitamin B6 deficiency is associated with microcytic anemia, electroencephalographic abnormalities, dermatitis with cheilosis (scaling on the lips and cracks at the corners of the mouth), glossitis (swollen tongue), depression, confusion, and weakened immune function, while its increase may lead to lack of muscle control or painful, skin lesions, heartburn and nausea.

Chickpeas, turkey, fish (tuna, salmon), bananas, potatoes, organ meats, whole grains, fortified cereals, and vegetables are vitamin B_6_-rich foods. Currently, adult males and females up to the age of fifty should consume 1.3 mg of vitamin B_6_ per day from meals or supplements.

The recommended daily allowance (RDA) is 1.7 mg for males and 1.5 mg for females over fifty^4,29,30^.

**Scheme 1:**
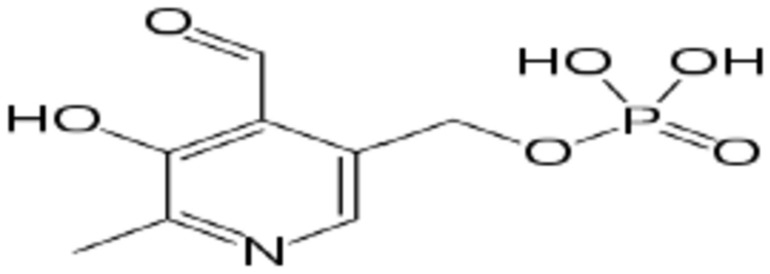
Vitamin B_6_.

Many searches investigated various analytical techniques for vitamin B_6_ (Scheme1) detection, such as spectrophotometric, fluorometric, electrophoresis, and HPLC. All these methods are time-consuming and need more flexibility and portability. Because of high sensitivity, simple procedure, good accuracy and fast response, electro-catalytic methods are currently receiving a lot of interest. Utilizing vanadium (III) Schiff base complex amended GCE for vitamin B_6_ determination was one of the electrochemical methods for vitamin B_6_ detection that had been the subject of several prior research^31^, using a copper (II) hexacyanoferrate (III) modified CPE to measure vitamin B_6_ in the production of medicinal chemicals^32^, voltametric sensor for ZrO_2_ nanoparticle/ionic liquids CPE in food samples for the immediate measurement of vitamin B_6_ and vitamin C^33^. As a result, the electrochemical sensors are perfect for monitoring the incorporation of biological compounds utilizing portable devices, mainly when this study uses inexpensive carbon paste electrodes loaded with simply prepared (electrodeposition), available, stable, highly reactive, low cost and well conducting nanoparticles such as copper nanoparticles (CuNs), which made its usage in numerous applications more easier, like catalysis, cooling fluid or conductive inks^34–38^.

This work aims to create a simple, highly sensitive, and inexpensive sensor for vitamin B_6_ detection which has been achieved compared to our previously published work that utilized iron nanoparticles^39^. A novel B_6_ sensor was created by simple electrodeposition of CuNPs onto the CPE surface compared to the complicated multistep preparation of iron nanoparticles in order to achieve the required modified electrode. Although both of the electrodes gave comparable detection limits values but our new CuNCP sensor shown much higher response toward Vitamin B_6_ as will be discussed upcoming. The utility of the CuNCP sensor was examined in real human urine samples and with drugs.

## Experimental

### Chemicals and reagents

Pure vitamin B_6_ (≥ 99.0%) from Pharaonia Pharmaceuticals (Egypt) was utilized to make the stock solution. Sigma-Aldrich's copper sulphate. To prepare 0.1 M supporting electrolytes, Sigma-Aldrich supplies ammonium hydroxide, sodium phosphate dibasic, sodium phosphate monobasic, and (sulphuric, phosphoric, and hydrochloric) acids. CPE was prepared using graphite microparticles (less than 50 μm) from Merck, Germany, and paraffin oil from Aldrich in the USA. The interference studies used caffeine powder from Alpha Chemika (Mumbai, India) and ascorbic acid from Merck. The following materials were acquired from MISR-Scientific Company: starch, glucose, urea, sucrose, and uric acid. l(+)-Ascorbic acid is obtained from Alpha-Chem, an Indian company.

### Apparatus

The electrochemical studies were conducted at room temperature utilizing a standard 25 mL three-electrode setup. The working electrode (WE) was either the bare electrode or the CuNCPE, while a saturated calomel electrode served as the reference electrode (RE), and a platinum rod functioned as the counter electrode (CE).

The electrochemical cell was linked to a computer-operated EC-Lab^®^ electrochemical software and a Bio-logic SAS model SP-150 potentiostat. Different electrochemical measurements, such as CV, EIS, CA, and DPV, could be done accurately with this setup. Using a sinusoidal voltage amplitude of 10 mV, electrochemical impedance spectroscopy (EIS) was performed between 100 mHz and 100 kHz in the frequency range. The EC-Lab^®^ program was utilized to perform the fitting and analysis of the data, employing the most optimal equivalent circuit model. Measurements were done at least three times to achieve a repeatable result.

The Adwa 1030 digital pH meter (Romania) was connected to measure the pH solution. SEM (Model Quanta 250 Field Emission Gun) was used to examine the morphology by attaching with an energy dispersive X-ray (EDX) Unit (FEI Company, Japan). Transmission electron microscopy (TEM) analysis was made using a JEM-1400 Electron Microscope (JEOL, Japan). The PAN-analytical X-Ray Diffraction equipment model X׳Pert PRO with secondary monochromator, operating at 45 kV and 35 mA with Cu-radiation (λ = 1.542 Å) and scanning speed of 0.04° s^−1^, was used for structural investigations. The diffraction lines located at 2θ values between 2° and 60°, the corresponding spacing (d, Å), and relative intensities (I/Io), were obtained. The diffraction charts and relative intensities are obtained and compared with ICDD files.

### CuNCPE preparations

**Scheme 2:**
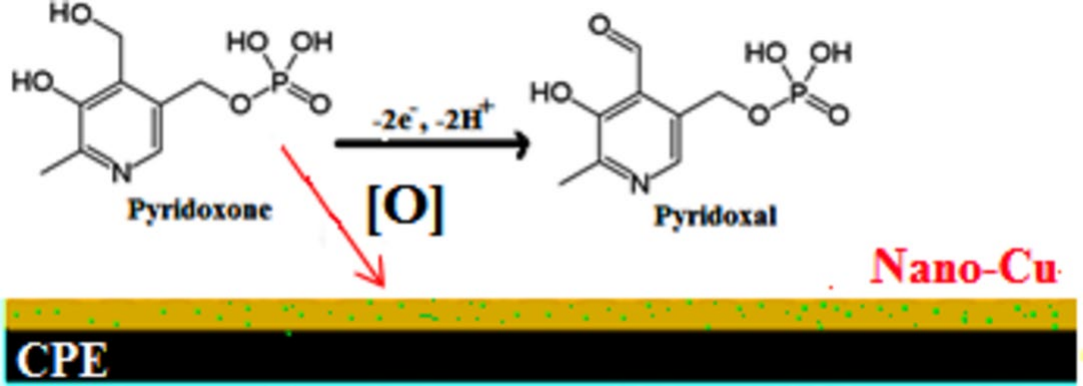
The suggested preparation steps of CuNCPE.

The CPE could be synthesized by first achieving a homogenous paste and manually combining 5.0 g of graphite powder and 3.0 mL of paraffin oil in a clean mortar for approximately 10 min. Then, fill a 3 mm-diameter hole at the end of a Teflon tube with this mixture. After that, CuNs were precipitated over the carbon paste electrode by immersing the electrode in the solution of 10^–4^ M CuSO_4_ and 0.25 M H_2_SO_4_ using a current density of 0.24 mA cm^−1^ for 15 min to achieve the CuNCPE as represented in Scheme 2^40,41^.

### Applications of sensor and preparing of real sample

Vitamin B_6_ level is properly measured using two samples (urine and multivitamin). First, add 0.25 mL of urine sample to 100 mL of 0.1 M PBS (pH 6.0) to create a ratio of 1:400 to eliminate any matrix effect. Then, take 22.5 mL of this mixture and add it gradually to the electrochemical cell using vitamin B_6_ until we reach 25 mL using the standard addition method. Second, one tablet was dissolved in 250 mL of distilled water to make the pharmacological samples. Next, 2.5 mL from the drug solution was mixed with 20 mL of 0.1 M PBS (pH 6.0) and assembled with the electrochemical cell utilizing vitamin B_6_ to make additions until reaching 25 mL.

## Results and discussion

### Characterizations of the surface

**Fig. 1:**
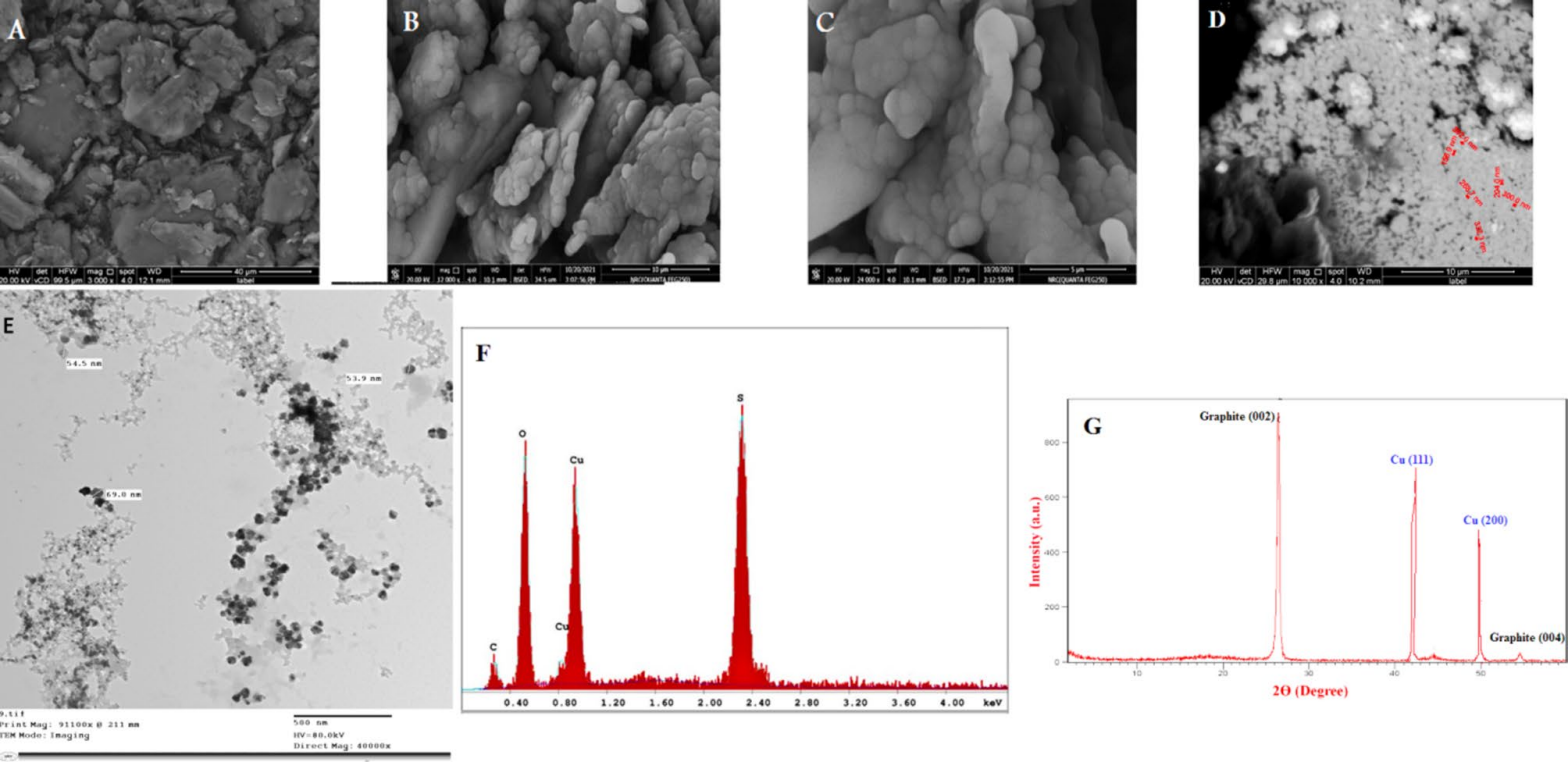
SEM images of (**A**) CPE and (**B**–**D**) CuNCPE with different magnifications. (**E**) Transmission electron microscopy (TEM) micrograph of CuNs (**F**) EDX spectrum of CuNs on CPE and (**G**) XRD pattern of CuNCPE.

Using SEM and EDX analysis techniques, the surface morphology of the amended electrode was examined, and the result was displayed in Fig. 1. Study of Fig. 1A, represented by CP, that looks like an opaque compact surface, which agrees with the high graphite powder compatibility in the paste formation with the mineral oil. The modified paste with CuNs was represented in Fig. 1B–D using different magnifications as shown, and it seems to be an aggregate of spherical nano-sized shapes like a cluster with large particle sizes as recorded on image Fig. 1D. Successful formation of CuNs onto the electrode surface, with spherical shape (and sizes of 54–70 nm) was evaluated from TEM examination as seen in Fig. 1E. The presence of copper, carbon, oxygen, and sulphur peaks is represented and confirmed using the EDX data of the CuNCPE displayed in Fig. 1F, which is a good representative of the paste formation.

The XRD pattern of the CuNCPE shows sharp diffraction lines at 26.6°, 42.5°, 50.2° and 54.5° (Fig. 1G). The two sharp diffraction lines located at 2θ = 42.5° (with d-spacing of 2.1 Å) and 50.2° (with d-spacing of 2.0 Å) correspond to the CuNs, while the other two peaks located at 2θ = 26.6° (with d spacing of 3.36 Å) and 54.5° (with d-spacing of 1.0 Å) are attributed to carbon, the other main constituent of the modified electrode. No additional lines originating from any other crystalline elements can be noticed, which indicates the high purity of the deposited CuNs and that their good crystallinity is supported by the sharpness of diffraction lines. The definite line broadening of the peaks suggests that CuNs are in the nanometer size range.

### Electrochemistry of vitamin B_6_ at CuNCPE modified sensor

**Fig. 2:**
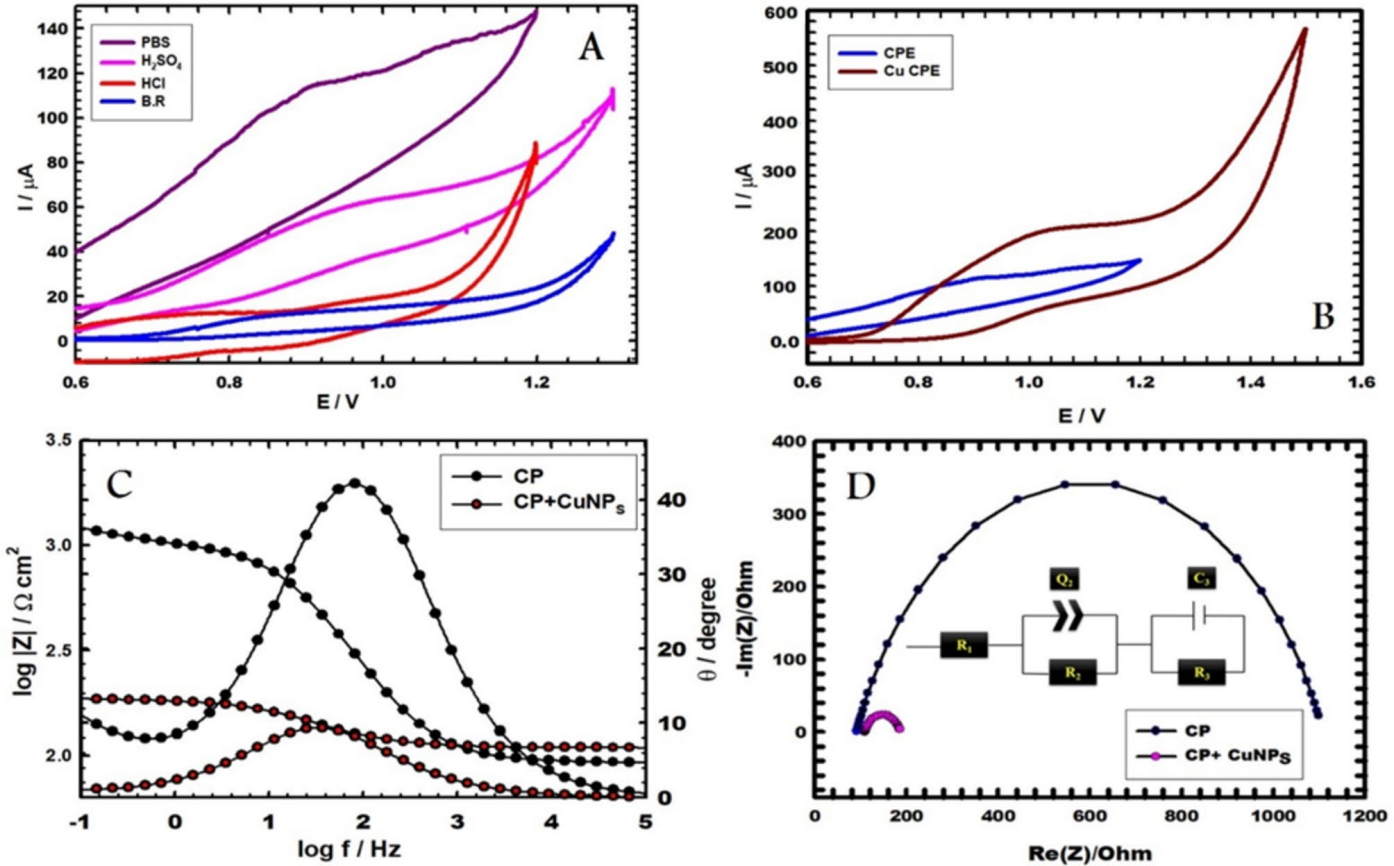
(**A**) CVs of bare CPE utilizing different supporting electrolytes with 1.0 mM vitamin B_6_ at scan rate 0.05 V s^−1^. (**B**) CVs of CPE and CuNCPE (in PBS, pH 5.0) with 1.0 mM vitamin B_6_. (**C**,**D**) Bode and Nyquist plots of CPE and CuNCPE (in PBS, pH 5.0) with 1.0 mM vitamin B_6_ (**D** inset: equivalent circuit).

Various kinds of buffer solutions like 0.1 M of each HCl, H_2_SO_4_, Britton-Robinson buffer (B-R), and phosphate buffer (PBS) were tested for use as supporting electrolytes, and that was because of enhancing the conditions of measurements. Figure 2A illustrates almost no peaks when using HCl and B-R buffer solutions. On the other hand, a wide and broad peak appears in the case of the H_2_SO_4_ buffer solution. However, PBS revealed a sharp peak, which makes it the appropriate operational solution. A voltammogram in the supporting electrolyte without vitamin B_6_ was also completed but it was nearly blank one with no peaks and coincided on the curve concerning B_R buffer.

To achieve a developed highly performed electrochemical sensor, how it performs towards the substance you want to measure compared with CPE is required. This substance was vitamin B_6,_ and the measurements were taken. The sensitivity and validity of CuNCPE were checked in the detection of vitamin B_6_ using the CV technique. Figure 2B demonstrates CVs of CPE and CuNCPE in 0.1 M of PBS at pH = 5.0 employed as supporting electrolytes, including 1.0 mM vitamin B_6_, using a scan rate of 0.05 V s^−1^. Currently, the vitamin B_6_ oxidation on CuNCPE is an irreversible process. A distinct oxidation peak can be seen at a peak potential of about 0.99 V, and the peak current is about 243.661 µA, approximately 1.266 times higher than the bare electrode's 192.4 µA.

The study of EIS was done at the peak potential (1.0 V), using Bode and Nyquist plots displayed in Fig. 2C and D, correspondingly and the results give a good confirmation for the results obtained from CVs, which declare that CuNCPE reaches an advanced electrocatalytic activity than bare CPE when it comes to the oxidation of 1.0 mM vitamin B_6_. The semi-circle-shaped Nyquist plot illustrates that the process depends on charge transfer resistance. With a 1% average error, the corresponding circuit's best attempt was to fit the data shown in Fig. 2D inset. Q_2_, the constant phase element CPE, is connected in parallel to R_2_, the outer layer resistance, while R_1_ denotes the solution resistance. The inner layer resistance, R_3_, and the double layer capacitance, C_3_, are connected in parallel^42,43^. Surface roughness causes an empirical exponent (α = 0 to 1) to be projected to inspect the departure from a capacitive idealist behaviour. The optimal capacitor is associated with α = 1, and the CPE in the Warburg component emerges at α = 0.5^44,45^. There is no straight line observed in the obtained curves which refer no diffusion behaviour is present. As well the empirical exponent values (α) wasn’t equal to 0.5, which is the value that may refer to the presence of Warburg component and diffusion. The fitting is completed applying EC-Lab^®^ software. A larger bare semicircle diameter than CuNCPE, implying a greater conductivity. These outcomes verify the maximum oxidation I_p_ acquired from CVs for CuNCPE electrode. The solution's resistance value, R_1_, is about a constant for both electrodes within the experimental error's limits. CuNCPE exhibits comparatively more significant capacitance values or lesser impedance values than BCPE, demonstrating a more conducting behaviour and confirming the uppermost oxidation peak current that was attained from CV's findings.

### Effect of pH

**Fig. 3:**
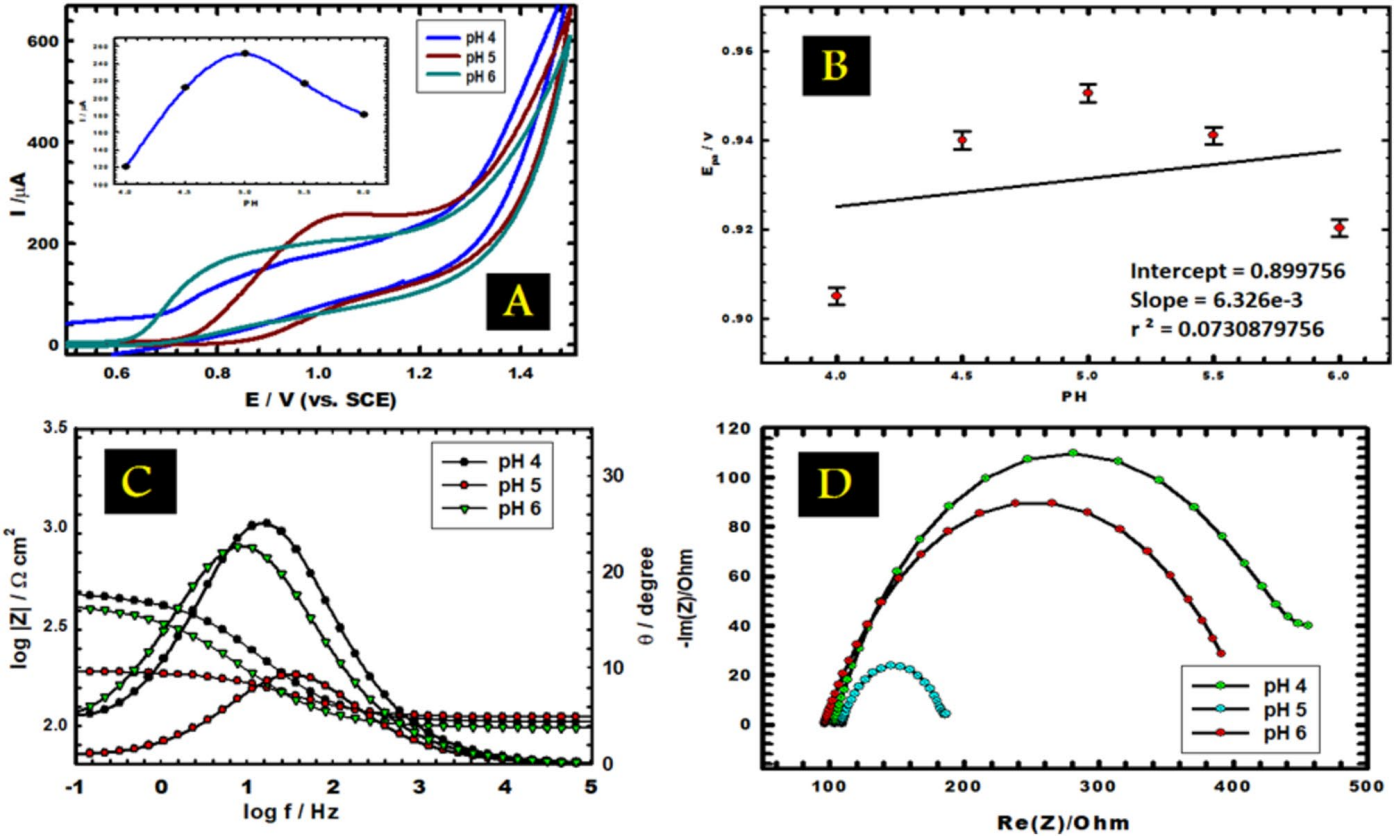
(**A**) CVs of vitamin B_6_ in 0.1 M PBS at different pH (4.0–6.0) at scan rate 0.05 V s^−1^. Inset, the variation of anodic peak current with pH. (**B**) The effect of pH on the anodic peak potential (**C**,**D**) Bode and Nyquist plots of 1.0 mM vitamin B_6_ at different pH values.

The impact of altering the solution's pH on the electro-oxidation of vitamin B_6_ was investigated at a concentration of 1.0 mM vitamin B_6_ in 0.1 M PBS (pH 4.0–6.0) at CuNCPE (Fig. 3A). The complicated scattering of species resultant from hydration and acid–base equilibria makes it clear that the pH affects vitamin B_6_ electro-oxidation. In an acidic medium (pH = 4), the expansion and eventual disappearance of the current peak can be accredited to vitamin B_6_ molecule protonation, which is electroactive only in its un-protonated form. An optimal peak appears as an increasing pH; functioning pH has been set to 5.0 for this work. The influence of pH on the current peak (Fig. 3A inset) demonstrates that the current raises when pH increases from 4.0 to 5.0 and then falls as pH upsurges, ensuring that vitamin B_6_ protonation processes in an acidic media and becomes electro-inactive. Additionally, when the pH solution increased, the peak current decreased and reached its virtual maximum at pH = 5.0. This is predictable as the vitamin is only in an anionic form in robust basic solutions (pH > 8.0) because of the deprotonation of the −OH group attached to the pyridinic ring. The peak current might decrease because of this. This decrease in anodic peak current in high basic pH could be ascribed to variations in the pace of reactivity of the electrode and the electrostatic interaction between CuNs and vitamin B_6_. Moreover, Nyquist and Bode plots were utilized to examine the effects of altering the pH of PBS (Fig. 3C and D). It tends to behave similarly to CV_S_. It demonstrates that pH = 5.0 maximum conductivity and minimum impedance are obtained with the lowest semicircle diameter. The anodic peak potential E_p_ change, as seen in Fig. 3B, illustrates how vitamin B_6_ oxidizes in response to pH. An increasing pH value was found to cause a negative shift in anodic E_p_, which corresponded to a linear relationship between pH and the potential peak in terms of the given equation:

1 $$ {\text{E}}_{{\text{p}}} \left( {\text{V}} \right) = 0.{899756 + }0.00{\text{6326 pH}}\;\;\;\;\left( {{\text{r}}^{{2}} = \, 0.0{73}} \right) $$

The proton-transfer process in vitamin B_6_ electro-oxidation was used to illustrate the anodic peak potential reliance on pH. Because of the complexity of the oxidation process, the slope (0.006326 V/pH) changes from the ideal Nernstian slope (= 0.059 V/pH) at 25 °C. This indicates the number of transported protons and electrons is not equal.

### Effect of scan rate

**Fig. 4:**
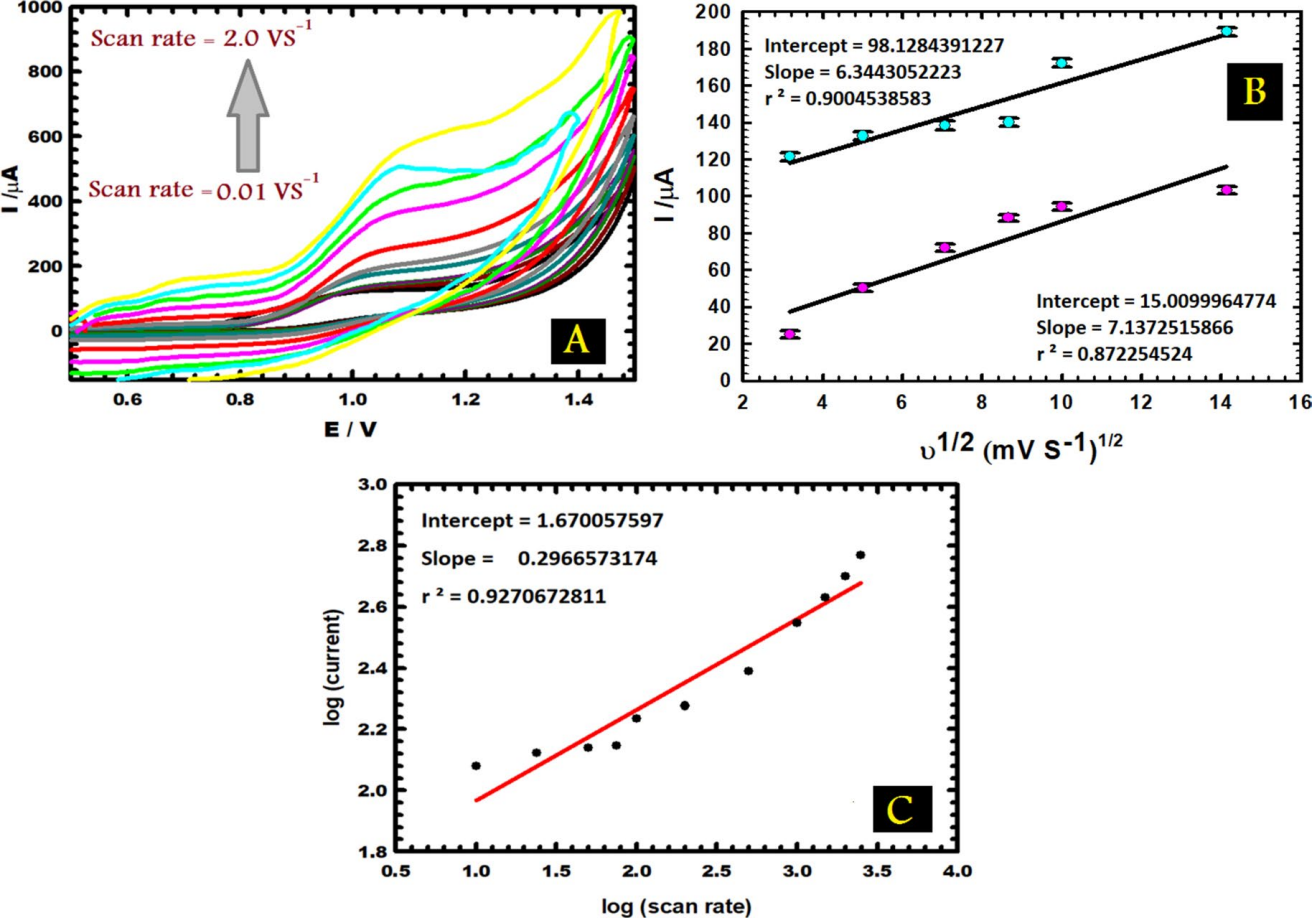
(**A**) CVs of 1.0 mM vitamin B_6_ (in PBS, pH 5.0) with different scan rates (0.01–2.0 V s^−1^). (**B**) The variation of the anodic peak current with the square root of scan rate (**C**) The variation of the logarithm of the anodic peak current with the logarithm of the scan rate.

The CV technique Fig. 4A illustrates the scan rate influence on the anodic peak current of 1.0 mM vitamin B_6_ in 0.1 M PBS. In a wide range, there was an increase in the scan rate (from 0.01 to 2.0 V s^−1^) followed by a rise in peak current; this caused the peak potential shifts to higher positive values, confirming the irreversible electrochemical oxidation process^46^. Figure 4B shows a linearity amongst the anodic peak current and the square root of the scan rate, which confirms the existence of a diffusion-controlled mechanism that could be signified by:

2 $$ {\text{I }}\left( {\upmu {\text{A}}} \right) \, = { 7}.{13725 }\upsilon^{{{1}/{2}}} + { 15}.00{999}; \;\; {\text{r}}^{{2}} = \, 0.{8722}\;\;{\text{for BCPE}} $$

3 $$ {\text{I }}\left( {\upmu {\text{A}}} \right) \, = { 6}.{3443}0{5 }\upsilon^{{{1}/{2}}} + { 98}.{128439}; \;\; {\text{r}}^{{2}} = \, 0.{9} \;\; {\text{for CuNCPE}} $$

Figure 4C illustrates a linear relation between log current against log scan rate with a slope of 0.296657, which designates the presence of a controlled mechanism; this means that through the electrode reaction, the active surface of CPEs has proven that they are an excellent environment for adsorbing particle and that was because of their surface passivation. After all, a mixed diffusion-adsorption mechanism was dominated.

**Scheme 3:**
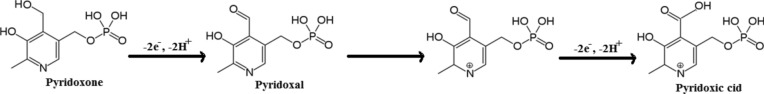
The suggested oxidation mechanism.

Scheme 3 shows that the pyridoxine electro-oxidation mechanism can form the aldehyde pyridoxal and then oxidize it to pyridoxic acid. Some cases show that detecting pyridoxal in its hemiacetal form was limited to an overall process. Alternatively, we might exclude the electroactive free aldehyde; however, this would necessitate a prior chemical change at the electrode within the voltammetric experiment's time frame^47^.

The diffusion coefficients of CPE and CuNCPE were assessed to be 0.613 and 0.57798 cm^2^ s^−1^, respectively, by inserting the electrode area, and the slope in Fig. 4B equals (2.69 × 10^5^) n^3/2^ACD^1/2^, based on the Randles–Sevcik equation. It is clear that adding CuN as a modifier has increased the active surface area and allowed vitamin B_6_ molecules to diffuse through the electrolyte.

### Calibration curve study

**Fig. 5:**
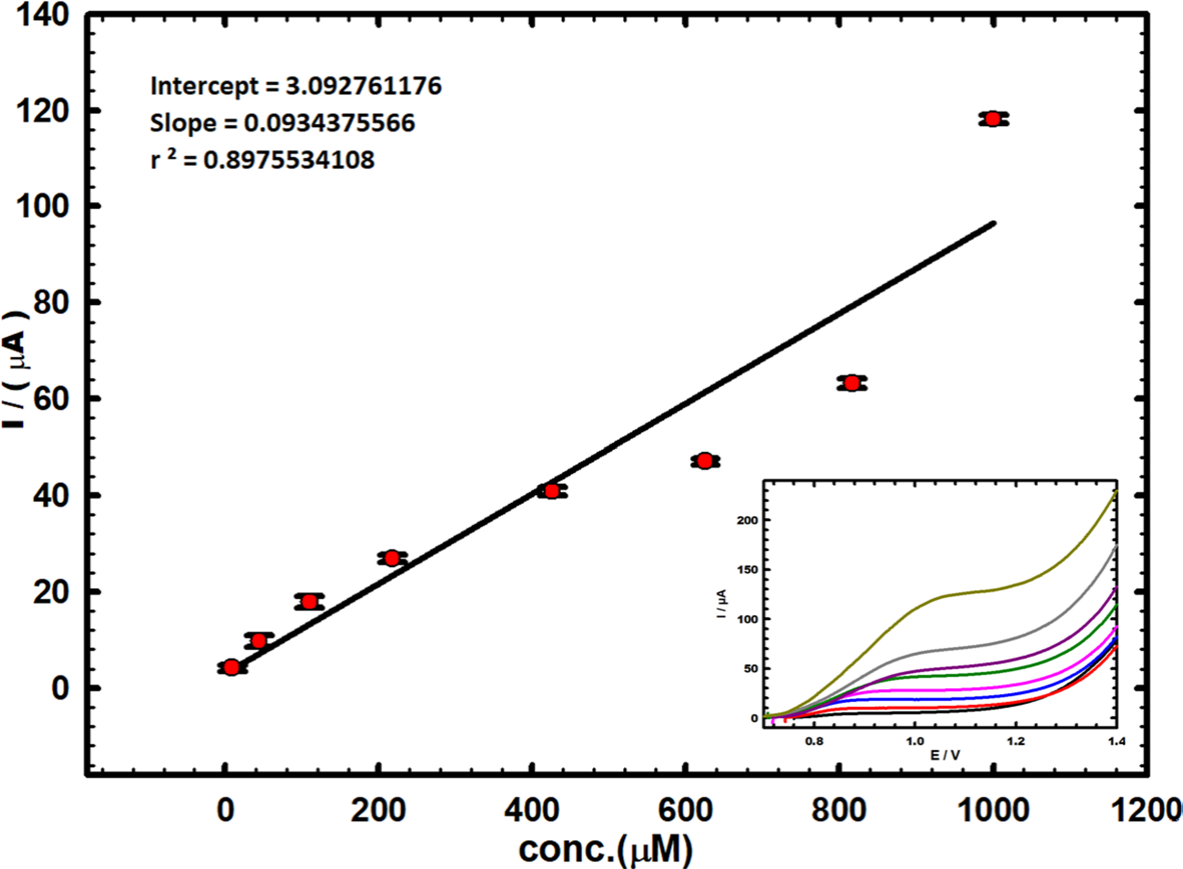
The calibration plot of vitamin B_6_ (in PBS, pH 5.0) utilizing CuNCPE. Inset: The recorded curves for successive vitamin B_6_ concentrations utilizing DPV at a step potential of 0.004 V, modulation amplitude of 0.025 V, and scan rate of 0.01 V s^−1^.

To ensure the validity and sensitivity of CuNCPE for the electrochemical determination of vitamin B_6_, Fig. 5 shows a linearity among the anodic current peak and different concentrations of vitamin B_6_ as follows:

4 $$ {\text{I }}\left( {\upmu {\text{A}}} \right) \, = { 3}.0{927 } + \, 0.0{\text{934 C }}\left( {\upmu {\text{M}}} \right) \;\; {\text{r}}^{{2}} = \, 0.{987} $$

Figure 5 inset displays the equivalent differential pulse patterns for vitamin B_6_. The samples were scanned at a rate of 0.01 V s^−1^, applying CuNCPE in 0.1 M of PBS (pH 5.0) with concentrations varying from 8.88 to 1000.0 µM. The limit of quantification (LOQ) and the limit of detection (LOD) were computed using the respecting formulas^48^:

5 $$ {\text{LOQ}} = {1}0\;\;{\text{s/m}} $$

6 $$ {\text{LOD }} = {3}\;{\text{s/m}} $$

The determined values of 107.0 and 32.12 µM validate the electrode's sensitivity. The reproducibility was well-ordered five times under the same standings as the suggested electrode by repeated measurements, producing a relative standard deviation (RSD) of 2.1%.

Table S1 compares the several electrochemical sensors that have already been discussed for vitamin B_6_ detection. In contrast to the modified electrode suggested in this work, those approaches involve the use of costly or hazardous chemicals in addition to being more complex to fabricate. Thus, with a comparatively low detection limit and strong selectivity, this approach has demonstrated its dependability and sensitivity for vitamin B_6_ detection.

### Samples analysis

To determine the utility of the new sensor by DPV, vitamin B_6_ was identified in real samples, including urine and drugs, by spiking the samples with standard vitamin B_6_ concentrations utilizing the standard addition method.

**Fig. 6:**
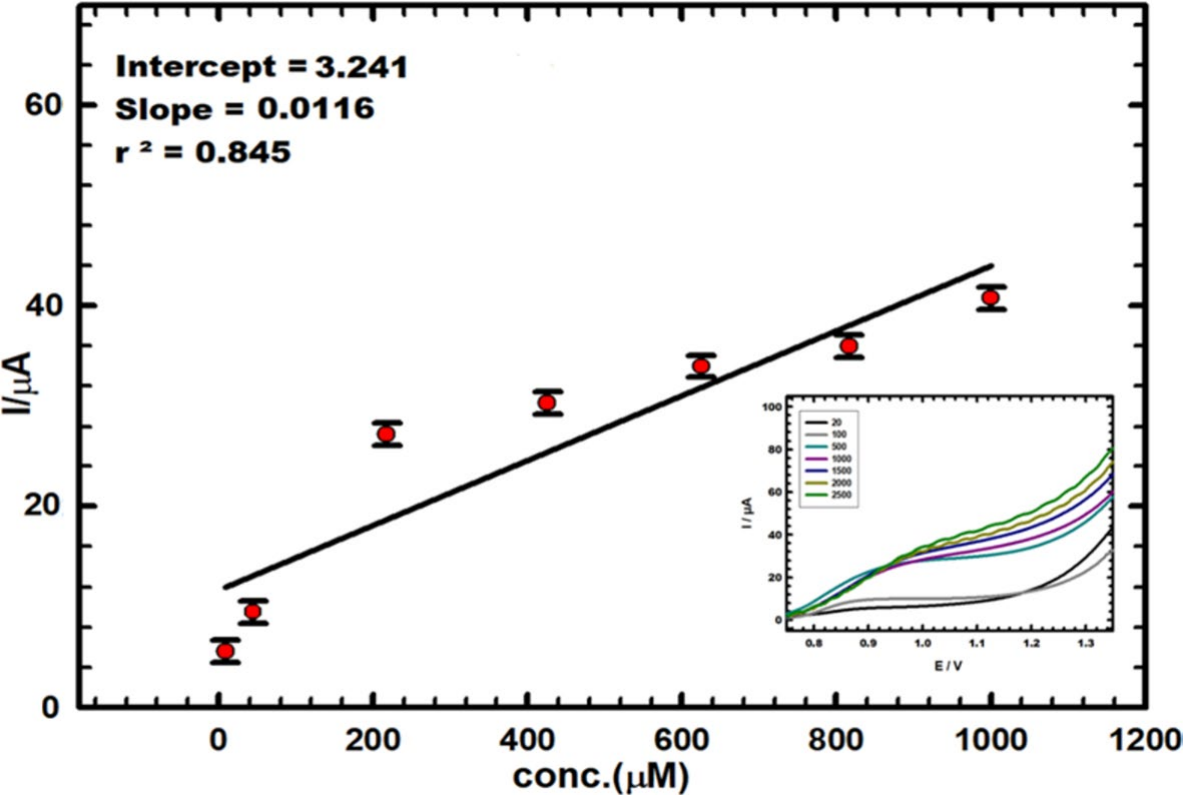
The calibration plot of vitamin B_6_ (in PBS, pH 5.0) utilizing CuNCPE in a real urine sample. Inset: the curves for successive vitamin B_6_ concentrations using DPV at step potential of 0.004 V, modulation amplitude of 0.025 V and scan rate 0.01 Vs^−1^.

The calibration curve for the CuNCP electrode using baby urine samples is displayed in Fig. 6 and results in a straight line in the concentration range of 8.0–1000.0 µM. The calibration curve equation was utilized to estimate the amount of vitamin B_6_ in urine samples: Ipa (µA) = 0.012C (M) + 3.241. The LOD was 25.0 µM, the LOQ was 83.3 µM, and the correlation value was r_2_ = 0.845. Table S2 demonstrates the recommended method of detecting vitamin B_6_ for four different concentrations on the curve with accuracy and precision; each was performed five times to ensure that the recommended methodology was validated in urine samples.

Table S2 displays the results, demonstrating that vitamin B_6_ can be estimated in pharmaceutical samples using the modified electrode, with satisfactory recoveries for each sample falling between 98.1 and 103.4% with RSDs of 1.2% to 3.9%. The amount of vitamin B_6_ in the real samples was determined by averaging five replicate measurements for each measurement of the oxidation current peak.

HPLC analysis was performed for Vitamin B_6_ sample and the results compared with that obtained from our proposed sensor to ensure excellent electrode response. The retention time was 4.43 min.

HPLC analysis was made on a chromatographic system model waters (Austria) equipped with UV 275 nm detector, 4.6-mm × 15-cm; packing L1 column and injection size equal to 10 µL.

For the analysis, the mobile phase consisted of Acetonitrile, tetrahydrofuran, and Buffer (25:20:955), adjusted with glacial acetic acid to a pH of 4.5 was used, at a flow rate of 1 mL/min.

**Fig. 7:**
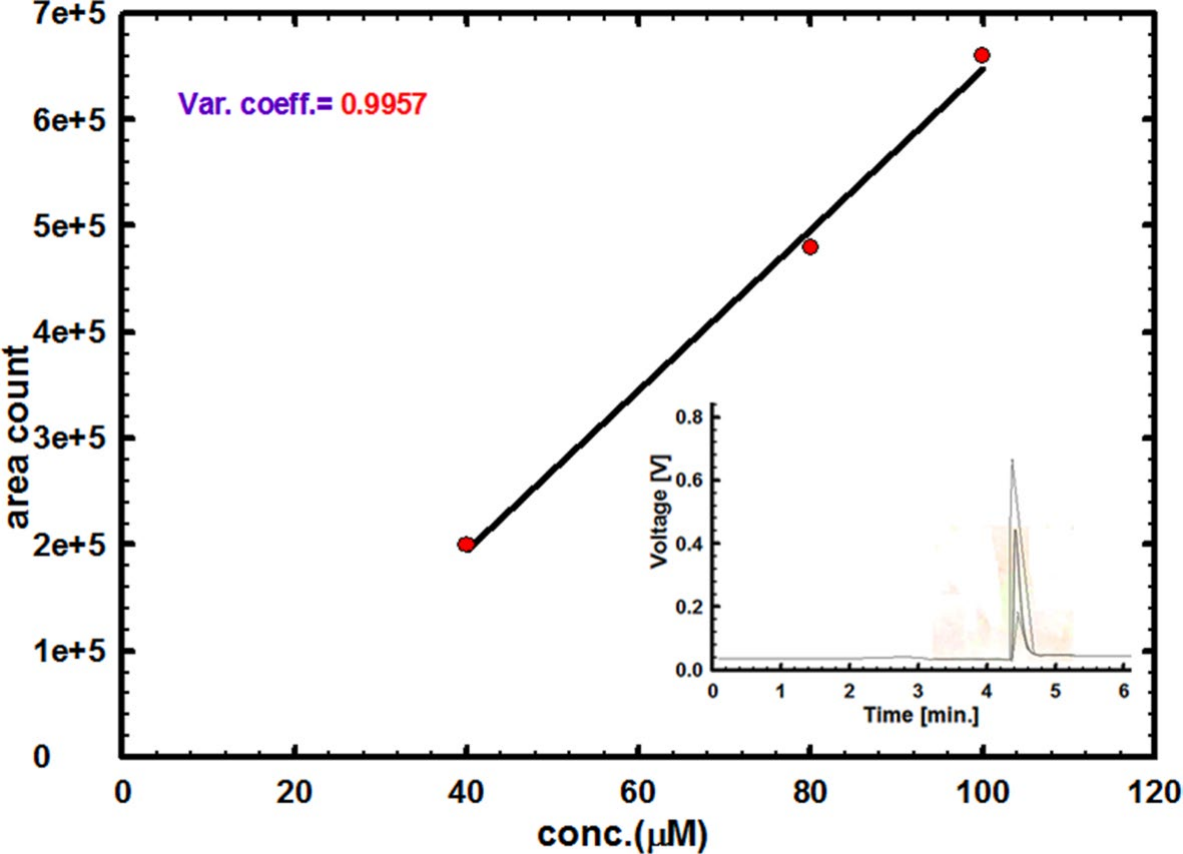
Calibration curve for Vitamin B_6_ using HPLC. Inset: the chromatograms of Vitamin B_6_ (t_R_/4.43), obtained by HPLC (column: 4.6-mm × 15-cm; packing L1; mobile phase/Acetonitrile, tetrahydrofuran, and Buffer (25:20:955), adjusted with glacial acetic acid to a pH of 4.5).

Typical chromatogram for Vitamin B_6_ obtained as illustrated in Fig. 7 inset. The mean value of peak area for each concentration was taken for the calibration curve at three different concentrations of Vitamin B_6_ with satisfied correlation coefficient factor of 0.995 and the slope of the curve used to calculate the LOD value which found to be 43.56 µM which is a comparable and little higher value to that is found in case of using our proposed sensor.

### Interfering materials, sensor selectivity, reproducibility and long-term stability

**Fig. 8:**
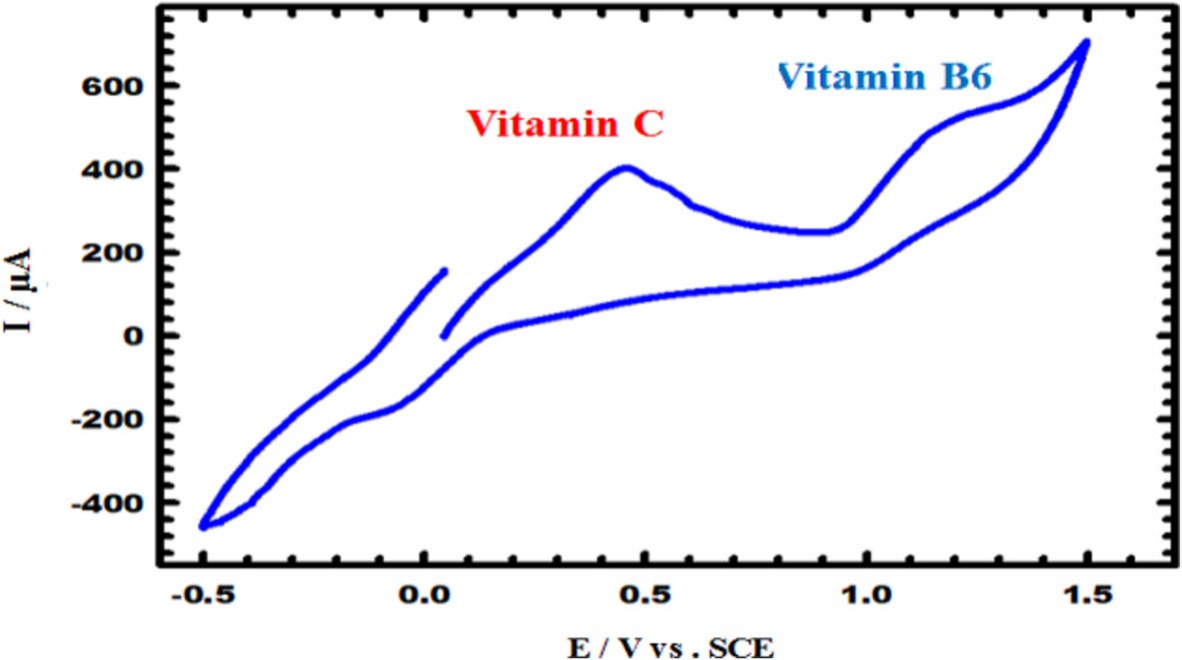
CV for the selectivity of CuNCPE to 5 × 10^–4^ M vitamin B_6_ in the existence of Ascorbic acid in 0.1 M PBS (pH 5.0) with scan rate 0.05 Vs^−1^.

Table S3 displays the results of studies on the voltammetric response with vitamin B_6_. The influence of various interfering substances on the determination of vitamin B_6_ by CuNCPE was evaluated by spiking a constant concentration of vitamin B_6_ (500 µM) with the same and doubled concentrations of different compounds, such as sucrose, starch, urea, glucose, uric acid, and vitamins, like ascorbic acid (vitamin C), thiamine hydrochloride (B_1_), riboflavin (B_2_), nicotinic acid (B_3_), pantothenic acid (B_5_), biotin (B_8_), folic acid (B_9_), and cyanocobalamin (B_12_). The results showed that, in the stated system (within the investigated potential range and in the selected supporting electrolyte), B_1_, B_2_, B_3_, B_5_, and B_8_ were not electroactive. Also, additional vitamins like ascorbic acid (vitamin C) were tested to choose the recommended method for vitamin B_6_. CV measurements were carried out under the same experimental settings, and without affecting the response of the vitamin B_6_ sensor, the sensor was capable of identifying both at different peak potentials of 1.12 V for vitamin B_6_ and 0.48 V for vitamin C (Fig. 8). This indicates that the sensors are highly selective.

To ensure the reproducibility of CuNCPE validation in terms of RSD, five consecutive voltammetric readings for 50 μM of vitamin B_6_ were recorded. The results showed no discernible fluctuation in vitamin B_6_ peak currents, with an RSD value of 1.9%. This guarantees the electrode being investigated is accurate.

The suggested procedure's long-term stability was evaluated by refrigerating CuNCPE at 4 ^◦^C for one week. After that, a voltammetric measurement for vitamin B_6_ was performed, and the results showed that the electrode had excellent storage stability, with a current response at 99.0% of the value achieved after fresh fabrication.

## Conclusions

Herein, we electrodeposited Cu nanoparticles onto a CPE surface to produce a new vitamin B_6_ sensor. Thanks to the increase in the electro-active area produced by Cu NPs, as established by the CV and EIS examinations, this sensor exhibited a brilliant vitamin B_6_ sensing routine. The electrochemical vitamin B_6_ response revealed a limit of detection of 32.12 × 10^–6^ M and a broad linear detection range (8.88 × 10^–6^–1.0 × 10^–3^ M). For determining vitamin B_6_, the sensor has good selectivity, sensitivity, and stability. Moreover, it has been shown that the sensor can precisely detect vitamin B_6_ in urine samples and Centrum multivitamins. We trust that CuNCPE could be helpful in the electrochemical detection of vitamin B_6_.

## Supplementary Information

Supplementary Information.

## Data Availability

The authors declare that the data supporting the findings of this study are available within the paper and its Supplementary Information files.

## References

[1] Muhammad, A. *et al.* Vitamins and minerals: Types, sources and their functions. In *Functional Foods and Nutraceuticals: Bioactive Components, Formulations and Innovations* 149–172 (2020).

[2] Schmidt, A., Schreiner, M. G. & Mayer, H. K. Rapid determination of the various native forms of vitamin B6 and B2 in cow’s milk using ultra-high performance liquid chromatography. *J. Chromatogr. A* **1500**, 89–95 (2017). 28420530 10.1016/j.chroma.2017.04.009

[3] Lee, J. H., Lauw, S. J. & Webster, R. D. The electrochemical reduction of carbon dioxide (CO_2_) to methanol in the presence of pyridoxine (vitamin B6). *Electrochem. Commun.* **64**, 69–73 (2016).

[4] Skarupski, K. A. *et al.* Longitudinal association of vitamin B-6, folate, and vitamin B-12 with depressive symptoms among older adults over time. *Am. J. Clin. Nutr.* **92**(2), 330–335 (2010). 20519557 10.3945/ajcn.2010.29413 PMC2904034

[5] Doll, H., Brown, S. U. S. A. N., Thurston, A. M. A. N. D. A. & Vessey, M. A. R. T. I. N. Pyridoxine (vitamin B6) and the premenstrual syndrome: A randomized crossover trial. *J. R. Coll. Gen. Pract.* **39**(326), 364–368 (1989). 2558186 PMC1711872

[6] Vutyavanich, T., Wongtra-ngan, S. & Ruangsri, R. Pyridoxine for nausea and vomiting of pregnancy: A randomized, double-blind, placebo-controlled trial. *Am. J. Obstet. Gynecol.* **173**(3), 881–884 (1995). 7573262 10.1016/0002-9378(95)90359-3

[7] Clayton, P. T. B 6-responsive disorders: A model of vitamin dependency. *J. Inherit. Metab. Dis.* **29**, 317–326 (2006). 16763894 10.1007/s10545-005-0243-2

[8] Lenze, E. J. *et al.* Onset of depression in elderly persons after hip fracture: Implications for prevention and early intervention of late-life depression. *J. Am. Geriatr. Soc.* **55**(1), 81–86 (2007). 17233689 10.1111/j.1532-5415.2006.01017.x

[9] Hvas, A.-M., Juul, S., Bech, P. & Nexø, E. Vitamin B6 level is associated with symptoms of depression. *Psychother. Psychosom.* **73**(6), 340–343 (2004). 15479988 10.1159/000080386

[10] Merete, C., Falcon, L. M. & Tucker, K. L. Vitamin B6 is associated with depressive symptomatology in Massachusetts elders. *J. Am. Coll. Nutr.* **27**(3), 421–427 (2008). 18838531 10.1080/07315724.2008.10719720 PMC2572855

[11] Chiang, E.-P.I., Bagley, P. J., Selhub, J., Nadeau, M. & Roubenoff, R. Abnormal vitamin B6 status is associated with severity of symptoms in patients with rheumatoid arthritis. *Am. J. Med.* **114**(4), 283–287 (2003). 12681455 10.1016/s0002-9343(02)01528-0

[12] Chiang, E.-P.I., Selhub, J., Bagley, P. J., Dallal, G. & Roubenoff, R. Pyridoxine supplementation corrects vitamin B6 deficiency but does not improve inflammation in patients with rheumatoid arthritis. *Arthritis Res. Ther.* **7**, 1–8 (2005). 16277693 10.1186/ar1839 PMC1297588

[13] Woolf, K. & Manore, M. M. Elevated plasma homocysteine and low vitamin B-6 status in nonsupplementing older women with rheumatoid arthritis. *J. Am. Diet. Assoc.* **108**(3), 443–453 (2008). 18313425 10.1016/j.jada.2007.12.001

[14] Huang, S. C., Wei, J. C. C., Wu, D. J. & Huang, Y. C. Vitamin B6 supplementation improves pro-inflammatory responses in patients with rheumatoid arthritis. *Eur. J. Clin. Nutr.* **64**(9), 1007–1013 (2010). 20571496 10.1038/ejcn.2010.107

[15] Christen, W. G., Glynn, R. J., Chew, E. Y., Albert, C. M. & Manson, J. E. Folic acid, vitamin B6, and vitamin B12 in combination and age-related macular degeneration in a randomized trial of women. *Arch. Intern. Med.* **169**(4), 335 (2009). 19237716 10.1001/archinternmed.2008.574 PMC2648137

[16] Sofi, F. *et al.* Low vitamin B6 and folic acid levels are associated with retinal vein occlusion independently of homocysteine levels. *Atherosclerosis* **198**(1), 223–227 (2008). 17945240 10.1016/j.atherosclerosis.2007.09.009

[17] Axer-Siegel, R. *et al.* Association of neovascular age-related macular degeneration and hyperhomocysteinemia. *Am. J. Ophthalmol.* **137**(1), 84–89 (2004). 14700648 10.1016/s0002-9394(03)00864-x

[18] Seddon, J. M., Gensler, G., Klein, M. L. & Milton, R. C. Evaluation of plasma homocysteine and risk of age-related macular degeneration. *Am. J. Ophthalmol.* **141**(1), 201–203 (2006). 16387004 10.1016/j.ajo.2005.07.059

[19] Seddon, J. M., Gensler, G., Klein, M. L. & Milton, R. C. C-reactive protein and homocysteine are associated with dietary and behavioral risk factors for age-related macular degeneration. *Nutrition* **22**(4), 441–443 (2006). 16530626 10.1016/j.nut.2005.12.004

[20] Malouf, R. & Sastre, A. A. Vitamin [B. sub. 12] for cognition. *Cochrane Database Syst. Rev.*10.1002/14651858.CD004394 (2003). 14584018 10.1002/14651858.CD004514

[21] Seshadri, S. *et al.* Plasma homocysteine as a risk factor for dementia and Alzheimer’s disease. *N. Engl. J. Med.* **346**(7), 476–483 (2002). 11844848 10.1056/NEJMoa011613

[22] Oulhaj, A. *et al.* Homocysteine as a predictor of cognitive decline in Alzheimer’s disease. *Int. J. Geriatr. Psychiatry* **25**(1), 82–90 (2010). 19484711 10.1002/gps.2303

[23] Lin, P.-T. *et al.* Low pyridoxal 5′-phosphate is associated with increased risk of coronary artery disease. *Nutrition* **22**(11–12), 1146–1151 (2006). 17045461 10.1016/j.nut.2006.08.013

[24] Vermeulen, E. G. J. *et al.* Effect of homocysteine-lowering treatment with folic acid plus vitamin B6 on cerebrovascular atherosclerosis and white matter abnormalities as determined by MRA and MRI: A placebo-controlled, randomized trial. *Eur. J. Clin. Investig.* **34**(4), 256–261 (2004). 15086356 10.1111/j.1365-2362.2004.01332.x

[25] Vermeulen, E. G. *et al.* Effect of homocysteine-lowering treatment with folic acid plus vitamin B6 on progression of subclinical atherosclerosis: a randomised, placebo-controlled trial. *Lancet* **355**(9203), 517–522 (2000). 10683000 10.1016/s0140-6736(99)07391-2

[26] Endo, N. *et al.* Antioxidant activity of vitamin B6 delays homocysteine-induced atherosclerosis in rats. *Br. J. Nutr.* **95**(6), 1088–1093 (2006). 16768830 10.1079/bjn20061764

[27] Bird, R. P. The emerging role of vitamin B6 in inflammation and carcinogenesis. *Adv. Food Nutr. Res.* **83**, 151–194 (2018). 29477221 10.1016/bs.afnr.2017.11.004

[28] Ueland, P. M., McCann, A., Midttun, Ø. & Ulvik, A. Inflammation, vitamin B6 and related pathways. *Mol. Aspects Med.* **53**, 10–27 (2017). 27593095 10.1016/j.mam.2016.08.001

[29] Katan, M. How much vitamin B6 is toxic?. *Nederlands tijdschrift voor geneeskunde.* **46**, 2545–2546 (2005). 16320662

[30] Spinneker, A. *et al.* Vitamin B6 status, deficiency and its consequences—An overview. *Nutricion hospitalaria* **22**(1), 7–24 (2007). 17260529

[31] Prasad, P. S., Kumar, S. P., Bharathi, K. & Narayanan, V. Determination of Vitamin-B6 by Vanadium (III) Schiff base complex modified GCE. *Mater. Today Proc.* **5**(2), 9026–9032 (2018).

[32] Mekonnen, A., Saini, R. C., Tadese, A. & Pal, R. Square wave voltammetric determination of pyridoxine in pharmaceutical preparations using cobalthexacyanoferrate modified carbon paste electrode. *J. Chem. Pharm. Res.* **6**(1), 544–551 (2014).

[33] Baghizadeh, A., Karimi-Maleh, H., Khoshnama, Z., Hassankhani, A. & Abbasghorbani, M. A voltammetric sensor for simultaneous determination of vitamin C and vitamin B_6_ in food samples using ZrO_2_ nanoparticle/ionic liquids carbon paste electrode. *Food Anal. Methods* **8**, 549–557 (2015).

[34] Dinesh, K. & Geetha, K. Synthesis and characterization of copper and copper oxide nanoparticles by thermal decomposition method. 321–327 (2014).

[35] Grouchko, M., Kamyshny, A., Ben-Ami, K. & Magdassi, S. Synthesis of copper nanoparticles catalyzed by pre-formed silver nanoparticles. *J. Nanoparticle Res.* **11**(3), 713–716 (2009).

[36] Usman, M. S. *et al.* Synthesis, characterization, and antimicrobial properties of copper nanoparticles. *Int. J. Nanomed.* 4467–4479 (2013). 10.2147/IJN.S50837 PMC3839804 24293998

[37] Tomaszewska, E. *et al.* Comparison of the effect of dietary copper nanoparticles with copper (II) salt on bone geometric and structural parameters as well as material characteristics in a rat model. *J. Trace Elem. Med. Biol.* **42**, 103–110 (2017). 28595781 10.1016/j.jtemb.2017.05.002

[38] Yoon, K. Y., Byeon, J. H., Park, J. H. & Hwang, J. Susceptibility constants of *Escherichia coli* and *Bacillus subtilis* to silver and copper nanoparticles. *Sci. Total Environ.* **373**(2–3), 572–575 (2007). 17173953 10.1016/j.scitotenv.2006.11.007

[39] Moustafa, A., El-Kamel, R. S., Abdelgawad, S., Fekry, A. M. & Shehata, M. Electrochemical determination of vitamin B6 (pyridoxine) by reformed carbon paste electrode with iron oxide nanoparticles. *Ionics* **28**(9), 4471–4484 (2022).

[40] Hosseini, J.-B., Raoof, S. & Ghasemi, Z. Gholami, Synthesis of Pt–Cu/poly (o-Anisidine) nanocomposite onto carbon paste electrode and its application for methanol oxidation. *Int. J. Hydrogen Energy* **40**, 292–302 (2015).

[41] Atta, N. F., Galal, A. & Azab, S. M. Electrochemical morphine sensing using gold nanoparticles modified carbon paste electrode. *Int. J. Electrochem. Sci* **6**, 5066–5081 (2011).

[42] Fekry, A. Impedance and hydrogen evolution studies on magnesium alloy in oxalic acid solution containing different anions. *Int. J. Hydrogen Energy* **23**, 12945–12951 (2010).

[43] Fekry, A. M. Electrochemical behavior of a novel nano-composite coat on Ti alloy in phosphate buffer solution for biomedical applications. *RSC Adv.* **24**, 20276–20285 (2016).

[44] Abdelrahman, E. & Essa, K. Three least-squares minimization approaches to interpret gravity data due to dipping faults. *Pure Appl. Geophys.* **172**(2), 427–438 (2015).

[45] Essa, K. S. & Elhussein, M. A new approach for the interpretation of magnetic data by a 2-D dipping dike. *J. Appl. Geophys.* **136**, 431–443 (2017).

[46] Bard, A. J. & Faulkner, L. R. Fundamentals and applications. *Electrochem. Methods* **2**, 482 (2001).

[47] Brunetti, B. & Desimoni, E. Voltammetric determination of vitamin B6 in food samples and dietary supplements. *J. Food Compos. Anal.* **2**, 155–160 (2014).

[48] Shehata, M., Fekry, A. M. & Walcarius, A. Moxifloxacin hydrochloride electrochemical detection at gold nanoparticles modified screen-printed electrode. *Sensors* **20**(10), 2797 (2020). 32423013 10.3390/s20102797 PMC7287685

