# Supplementary material for: Electrochemical sensing of vitamin B6 (pyridoxine) by adapted carbon paste electrode
Source: Sci Rep. 2024 Sep 20;14:21972. doi: 10.1038/s41598-024-71341-2 (PMC11415389; doi:10.1038/s41598-024-71341-2)
Supplement: Supplementary file 1 — Supplementary Information. [file 41598_2024_71341_MOESM1_ESM.docx]

**Supplementary Materials**

**Table S1. Comparison of CuNCPE to other analytical and electrochemical techniques.**

| **Refrences** | **LDR (M)** | **LOD (μM)** | **Technique** | **Sensor Name** |
| --- | --- | --- | --- | --- |
| **[49]** | **2.5×10^-6^-1.0×10^-3^** | **1.0** | Micellar electrokinetic chromatography | Amperometric electrochemical detection |
| **[50]** | **7.5×10^-5^-7.5×10^-2^** | **0.8** | CV | Electrochemically pretreated glassy carbon electrode |
| **[51]** |  | **37.0** | CV | Glassy Carbon Electrode |
| **[52]** |  | **2.81** | CV | pencil graphite electrode |
| **[53]** |  | **0.05** | DPV | Glassy Carbon Electrodes |
| **[54]** |  | **0.03** | HPLC-RP method | High performance liquid chromatography |
| **[55]** |  | **0.42** | DPV | MWCNTs-Mn^III^salen |
| **[56]** | **2.4×10^-6^-1.7×10^-5^** | **0.013** | HPLC | Coulometric electrochemical and ultraviolet detection |
| **[57]** | **1.2×10^-5^-6.9×10^-4^** | **0.41** | CV | Multi wall carbon nanotube modified carbon-ceramic electrode |
| **[58]** | **1.3×10^-6^-1.3×10^-5^** | **0.35** | CV | Glassy Carbon Electrode Modified with Chromium (III) Hexacyanoferrate(II) |
| **[59]** | **3×10^-7^-2×10^-4^** | **0.1** | DPV | Glassy Carbon Electrode |
| **[39]** | **8.88×10^-6^-1×10^-3^** | **9.06** | DPV | Carbon based electrode with ION |
| **This work** | **8.88×10^-6^-1×10^-3^** | **32.12** | DPV | Carbon based electrode with CuN |

**Table S2. Recoveries of vitamin B6 in urine & pharmaceutical samples at pH 5.0. RSD (%) was calculated for the average of five experiments.**

| Vitamin B_6_ | Added  (μM) | Expected  (μM) | Found  (μM) | Recovery  (%) | RSD  (%) |
| --- | --- | --- | --- | --- | --- |
| Urine Sample | | | | | |
|  | **0** | **-** | **50** | **-** | **1.3** |
|  | **30** | **80** | **78.5** | **98.1** | **1.4** |
|  | **60** | **110** | **114.0** | **103.6** | **2.6** |
|  | **90** | **140** | **143.0** | **102.1** | **2.2** |
| Pharmaceutical Sample ( Centrum multivitamin ) | | | | | |
|  | **0** | **-** | **40** |  | **3.9** |
|  | **10** | **50** | **48** | **96.0** | **1.2** |
|  | **20** | **60** | **62** | **103.3** | **2.3** |
|  | **30** | **70** | **70** | **100.0** | **1.8** |
|  | **40** | **80** | **79** | **98.8** | **2.1** |

| **Table S3. The effect of interfering materials on sensing of vitamin B_6_ by CuNCPE was calculated for the average of five measurements.** | | |
| --- | --- | --- |
| **Interfering material** | **Relative sensor response (%)** | |
|  | **1:1** | **1:2** |
| **Glucose** | **99.0** | **99.5** |
| **Sucrose** | **98.6** | **99.2** |
| **Starch** | **99.9** | **100.0** |
| **Urea** | **98.7** | **99.3** |
| **Uric acid** | **99.4** | **99.9** |
| **L-(+)-Ascorbic acid** | **99.7** | **99.9** |
| **Thiamine hydrochloride(B_1_), Riboflavin (B_2_), nicotinic acid (B_3_), pantothenic acid (B_5_), biotin (B_8_), folic acid (B_9_), and cyanocobalamin (B_12_)** | **100.0** | **100.0** |

[49] Hu, Q., T. Zhou, L. Zhang, H. Li, and Y. Fang. "Separation and determination of three water-soluble vitamins in pharmaceutical preparations and food by micellar electrokinetic chromatography with amperometric electrochemical detection." *Analytica chimica acta* 437, no. 1 (2001): 123-129. [Table 1S].

[50] Gu, H.-Y., A.-M. Yu, and H.-Y. Chen. "Electrochemical behavior and simultaneous determination of vitamin B2, B6, and C at electrochemically pretreated glassy carbon electrode." *Analytical letters* 34, no. 13 (2001): 2361-2374.

[51] Teixeira, M. FS, G. Marino, E. R. Dockal, and É. TG Cavalheiro. "Voltammetric determination of pyridoxine (Vitamin B6) at a carbon paste electrode modified with vanadyl (IV)–Salen complex." *Analytica chimica acta* 508, no. 1 (2004): 79-85.

[52] David, I. Gabriela, M.-Al. Florea, O. G. Cracea, D. E. Popa, M. Buleandra, E. E. Iorgulescu, V. David, I. A. Badea, and A. A. Ciucu. "Voltammetric determination of B 1 and B 6 vitamins using a pencil graphite electrode." *Chemical Papers* 69 (2015): 901-910.

[53] Nie, Tao, Jing-Kun Xu, Li-Min Lu, Kai-Xin Zhang, Ling Bai, and Yang-Ping Wen. "Electroactive species-doped poly (3, 4-ethylenedioxythiophene) films: Enhanced sensitivity for electrochemical simultaneous determination of vitamins B2, B6 and C." *Biosensors and Bioelectronics* 50 (2013): 244-250.

[54] Markopoulou, C. K., K. A. Kagkadis, and J. E. Koundourellis. "An optimized method for the simultaneous determination of vitamins B1, B6, B12, in multivitamin tablets by high performance liquid chromatography." *Journal of pharmaceutical and biomedical analysis* 30, no. 4 (2002): 1403-1410.

[55] Sonkar, P. Kumar, V. Ganesan, S. K. Sen Gupta, D. Kumar Yadav, R. Gupta, and M. Yadav. "Highly dispersed multiwalled carbon nanotubes coupled manganese salen nanostructure for simultaneous electrochemical sensing of vitamin B2 and B6." *Journal of Electroanalytical Chemistry* 807 (2017): 235-243.

[56] Marszałł, M. Leszek, A. Lebiedzińska, W. Czarnowski, and P. Szefer. "High-performance liquid chromatography method for the simultaneous determination of thiamine hydrochloride, pyridoxine hydrochloride and cyanocobalamin in pharmaceutical formulations using coulometric electrochemical and ultraviolet detection." *Journal of Chromatography A* 1094, no. 1-2 (2005): 91-98.

[57] Habibi, B., M. Jahanbakhshi, and M. Abazari. "A modified single-walled carbon nanotubes/carbon-ceramic electrode for simultaneous voltammetric determination of paracetamol and caffeine." *Journal of the Iranian Chemical Society* 11 (2014): 511-521.

[58] Cottica, S. M., J. Nozaki, H. S. Nakatani, C. C. Oliveira, N. E. de Souza, and J. V. Visentainer. "Voltammetric determination of pyridoxine (vitamin B6) in drugs using a glassy carbon electrode modified with chromium (III) hexacyanoferrate (II)." *Journal of the Brazilian Chemical Society* 20 (2009): 496-501.

[59] Wu, Y., and F. Song. "Voltammetric Investigation of Vitamin B~ 6 at a Glassy Carbon Electrode and Its Application in Determination." *BULLETIN-KOREAN CHEMICAL SOCIETY* 29, no. 1 (2008): 38.
